# Molecular Mechanism of MLC 901 in Acute Ischemic Stroke: A Review

**DOI:** 10.1155/srat/8973724

**Published:** 2025-10-24

**Authors:** Ilsa Hunaifi, Andi Kurnia Bintang, Jumraini Tammasse, Isra' Wahid, Mochammad Hatta, Andi Asadul Islam, Andi Alfian Zainuddin, Paulus Sugianto

**Affiliations:** ^1^Department of Neurology, Medical Faculty and Health Sciences University of Mataram, Mataram, Indonesia; ^2^Department of Neurology, Hasanuddin University, Makassar, Indonesia; ^3^Department of Parasitology, Hasanuddin University, Makassar, Indonesia; ^4^Department of Microbiology, Molecular Biology and Immunology Laboratory, Hasanuddin University, Makassar, Indonesia; ^5^Department of Neurosurgery, Hasanuddin University, Makassar, Indonesia; ^6^Department of Public Health, Hasanuddin University, Makassar, Indonesia; ^7^Department of Neurology, Airlangga University, Surabaya, Indonesia

## Abstract

Ischemic stroke is the leading cause of stroke all around the world. Ischemic stroke can cause severe and irreversible damage to the brain. Brain neuroprotection is a promising treatment strategy. Moleac (MLC) 901, a traditional Chinese medicine, is one alternative adjunctive therapy that enhances neuroprotection. The molecular mechanism of MLC 901 in reducing brain damage in stroke needs to be further explored. Therefore, a review was conducted. MLC 901 effectively improves cognitive function and aids in poststroke recovery by promoting neuroprotection and neuroplasticity in neurons that have suffered ischemic damage. It also increases blood supply to the brain. Studies have shown that MLC 901 operates through complex pathways, particularly by stimulating brain-derived neurotrophic factor (BDNF) expression, facilitating neurogenesis, promoting cell proliferation, and aiding in neuronal growth. These mechanisms collectively contribute to its neuroprotective effects by helping neurons survive, repairing brain tissue, and enhancing functional recovery after a stroke. Ischemic stroke induces a cascade leading to apoptosis. MLC 901 has neuroprotective and neuroplasticity effects by stimulating cell proliferation, synaptogenesis, and neuronal growth by various pathways, thereby inhibiting apoptosis and inflammation in acute ischemic stroke. MLC 901 reduces brain damage, improves motor function, and increases survival rate in acute ischemic stroke.

## 1. Introduction

Stroke is the second leading cause of disability and death worldwide. The incidence of ischemic stroke is rising globally, with a mortality rate of 21% and a disability rate of 18%. Acute ischemic stroke incidence increases by 5.3% annually [1, 2]. Ischemic stroke dominates all stroke events with a prevalence of 87% [3–5]. Ischemic stroke is a pathological condition characterized by the sudden occlusion of cerebral blood vessels, resulting in various neurological deficits [6]. Clinical manifestations of stroke include motor, verbal, and visual abnormalities like weakness, numbness, and aphasia [3]. Ischemic stroke often leads to devastating and irreversible brain damage. The pathological mechanisms underlying ischemic stroke are multifaceted and influenced by several factors, including the duration and intensity of cerebral ischemia [7].

Biochemical events in the ischemic cascade include energy failure, excitotoxicity, mitochondrial dysfunction, and apoptosis [8–10]. Failure of energy metabolism, glutamate excitotoxicity, and increased Ca^2+^ occur within 24 h of the onset of the stroke, accompanied by the formation of free radicals; apoptosis and necrosis also occur within hours of ischemia [9, 10]. Therefore, early treatment after ischemic stroke onset is vital for preventing severe complications and improving prognosis [6, 11].

The American Heart Association (AHA) standard treatment for ischemic stroke is thrombolysis with alteplase, administered within 4.5 h [12, 13]. Research in the United States indicates that only 2%–5% of patients receive thrombolysis treatment, primarily due to delayed arrival to medical services [14]. Thus, developing effective treatment strategies for neuroprotection against cerebral ischemic/reperfusion injuries is crucial to prevent cell death. Natural products play a significant role in disease prevention and therapy. Various natural products enhance neuroprotection in stroke through diverse biological activities and enzymatic mechanisms [10].

Herbal medicine has the potential to enhance microcirculation in the brain, decrease oxidative stress, and regulate microglial polarization [15]. Moleac (MLC) 901, a traditional Chinese medicine containing nine herbal components and various active compounds, is effective in improving cognitive function and poststroke recovery through the promotion of neuroprotective effects and neuroplasticity in neurons suffering from ischemic damage, as well as increased blood supply to the brain [16–18]. However, the molecular mechanism of MLC 901 in providing neuroprotective effects postischemic stroke is still limited. Therefore, this review focuses on the molecular mechanisms of MLC 901 in acute ischemic stroke.

## 2. Materials and Methods

This literature review used a variety of sources such as journal articles and official guidelines. Article searching is done in online journal publication sites such as the National Center for Biotechnology Information (NCBI), PubMed, PubMed Central, Frontiers in Microbiology, and Google Scholar. The keywords that were used were “MLC 901,” “Ischemic Stroke,” and “Biochemical Events of Stroke.”

## 3. Results and Discussion

### 3.1. Molecular Mechanism of Acute Ischemic Stroke

Ischemic stroke is a neurological dysfunction caused by focal, spinal, and retinal cerebral infarction as evidenced by cell death in the brain, spinal cord, and retina according to vascular distribution based on imaging or pathology, persisting ≥ 24 h or death and other causes have been excluded [19]. Two distinct damaged areas of brain tissue, a peripheral ischemic penumbra or peri-infarct zone that gets collateral blood flow and a central ischemic core, are the hallmarks of an ischemic stroke [4]. Necrotic cell death in the vicinity of the ischemic core leaves it metabolically, electrically, and functionally inert [20]. Between the ischemic core and normal, healthy tissue lies an area called the ischemic penumbra, where neurons are both electrically and functionally disrupted but also metabolically active [21].

**Figure 1:**
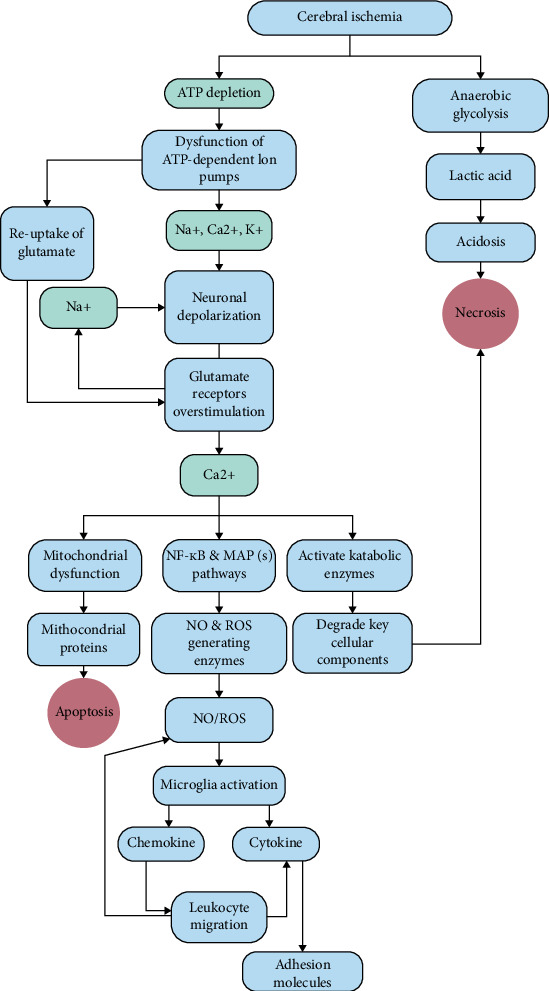
Diagram of the molecular mechanism in cerebral ischemia. When a stroke occurs, there is a reduction in blood flow, resulting in inadequate delivery of oxygen and glucose to the brain. As a result, ATP production via oxidative phosphorylation slows or stops, causing bioenergetic failure. Neuronal depolarization is the outcome of extensive ATP-dependent ion pump malfunction brought on by bioenergetic failure during a stroke. As a result, voltage-gated Ca^2+^ channels will open, resulting in overstimulation of glutamate release. Furthermore, energy failure will impair glutamate transporter ATP-dependent reuptake. Additional Ca^2+^ and Na^+^ ions will enter the cells as a result of glutamate buildup activating glutamate receptors on nearby neurons. Extreme necrotic excitotoxic neuronal cell death will result from these metabolic alterations unless and until the energy source is restored. Moreover, anaerobic glycolysis brought on by a reduction in oxygen availability may lead to an increase in lactate production and accumulation in the ischemic tissue. Acidotoxicity and necrotic brain cells arise from an accumulation of lactate, which lowers intracellular pH (acidosis). The authors modified this figure from their prior article [22]. Increased Ca^2+^ ion concentrations in the cytosol of neurons can activate catabolic enzymes as well as ROS and NO-generating enzymes, resulting in mitochondrial dysfunction. ROS can cause apoptosis by activating the nuclear factor kappa B (NF-*κ*B) and MAPK(s) signaling pathways. ROS can activate microglial cells, resulting in higher production of proinflammatory cytokines. This can cause cell damage by increasing the expression of cell adhesion molecules on endothelial cells and leukocytes, allowing leukocytes to infiltrate during reperfusion and release more ROS and proinflammatory cytokines. Moreover, microglial cells have the ability to generate chemokines, which might guide other leukocyte migration toward the ischemic area and result in further harm. The authors' earlier review served as the model for this particular figure [24]. ATP: adenosine triphosphate, NO: nitric oxide, ROS: reactive oxygen species, NF-*κ*B: nuclear factor kappa-light-chain-enhancer of activated B cells, MAP: mitogen-activated protein, Na^+^: natrium, Ca^2+^: calcium, K^+^: kalium.

The degree and duration of ischemia are two important parameters that influence the pathogenic processes of ischemic brain damage [22]. Recanalization, the primary goal of acute reperfusion treatment for the occluded artery, followed by restoration of blood flow, may be detrimental and initiate reperfusion injury, which could exacerbate the extent of ischemic injury and delay recovery efforts [23]. The ischemia cascade and reperfusion injury are characterized by biochemical processes that lead to cell death from necrosis or apoptosis (Figure 1).

The first insult caused by cerebral ischemia is bioenergetic failure as a result of oxygen and glucose deprivation, which results in decreased adenosine triphosphate (ATP) production in the mitochondria [25, 26]. Lack of ATP results in failure of ATP-dependent ion pumps, such as Ca^2+^-ATPase and plasma membrane Na^+^/K-ATPase, which causes an excessive inflow of Ca^2+^ and Na^+^ ions and an outflow of K− ions. Cytotoxic edema can arise from osmotic water transport into the cell via aquaporins, which is facilitated by elevated Na^+^ ion levels in neurons and glial cells [27, 28]. Another early factor in neuronal degeneration is the increased production of hydrogen ions (H^+^) in the environment brought on by anaerobic glycolysis. Lactic acidosis, which arises from anaerobic glycolysis, causes acidotoxicity and lowers the pH of the extracellular environment by raising the concentration of H^+^ ions [29].

Acidosis promotes neuronal damage, decreased astrocyte function, free radical formation, failure of ATP production in mitochondria, impaired protein synthesis, failure of intracellular signal transduction, and induction of deoxyribonucleic acid (DNA) damage and proinflammatory cytokine receptors [30, 31]. The mechanism of acidosis-related brain cell death is centered on the role of ion channels that are sensitive to changes in pH. One of the acid-sensitive ion channels is acid-sensing ion channel 1a (ASIC1a). Neuronal ASIC1a becomes a nonvoltage gate for Ca^2+^ inflow, resulting in intracellular Ca^2+^ accumulation and brain cell death. In addition, N-methyl-D-aspartate (NMDA) receptors increase the role of ASIC1a, resulting in intracellular Ca^2+^ influx. The role of ASIC1a in the necrosis process is through the interaction between cell death regulators on serine/threonine kinase receptors with receptor interaction protein 1 (RIPK1) during acidosis [31, 32].

Decreased ATP levels during ischemia also disrupt glutamate transporters' ability to reuptake glutamate in neurons and glia, leading to synaptic glutamate buildup that triggers kainate, NMDA, and *α*-amino-3-hydroxy-5-methyl-4-isoxazole propionic acid (AMPA) receptors [33]. Overactivation of glutamate receptors causes Ca^2+^ and Na^+^ influx into the cytosol. Simultaneously, the elevation in Ca^2+^ influx triggers a sequence of signaling reactions leading to metabolic disruptions referred to as excitotoxicity [34, 35]. Catabolic enzymes, in particular endonucleases, which cleave DNA to cause apoptosis, and calpains, which hydrolyze cytoskeletal, membrane-associated, and signaling proteins, are activated by Ca^2+^ levels in the cytoplasm and mitochondria [36]. Glutamate release and Ca^2+^ influx generate multiple consequences and changes in astrocytes, oligodendrocytes, and microglia. In astrocytes, there is increased expression of NMDA receptors; in oligodendrocytes, activation of AMPA and kainate receptors results in oligodendrocyte death and myelin damage; in microglia, glutamate activation results in increased release of tumor necrosis factor-alpha (TNF-*α*), nitric oxide (NO), and interleukin (IL)-1 [37].

**Figure 2:**
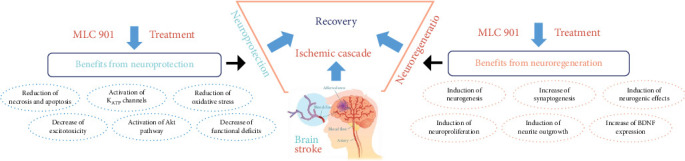
Neuroprotection and neuroregeneration effects of MLC 901 in stroke.

Oxidative stress also occurs in ischemic stroke, which is caused by the rise in the production of reactive oxygen species (ROS), and ultimately causes tissue damage [22]. The production of ROS is initiated by increasing the amount of cytosolic and mitochondrial Ca^2+^ [38]. The buildup of Ca^2+^ ions into the mitochondrial matrix via the mitochondrial calcium uniporter reduces the transmembrane potential, forming transition pores that allow electron leakage and excessive superoxide production [39]. Increased superoxide production frequently results in the production of other ROS such as hydrogen peroxide (H_2_O_2_), hydroxyl radicals (OH−), and peroxynitrite (ONOO−, produced by the interaction of superoxide with NO). ROS cause tissue damage by oxidizing macromolecules such as nucleic acids, proteins, carbohydrates, and lipids [40, 41]. ROS trigger an inflammatory response and release proinflammatory mediators, cytokines, and chemokines that stimulate leukocyte adhesion molecules and cytokine gene expression through the initiation of transcription factors such as nuclear factor kappa-*β* (NF-k*β*) [42] (Figure 2).

The first inflammatory cells to respond after cerebral ischemia begins are local brain microglia, which initiate an inflammatory response in stroke. Studies showed that the microglia in the penumbra region are the leading cause of necrotic cells [43]. Ischemic stroke also caused alterations in blood–brain barrier (BBB) permeability and the structure of vessel wall basal lamina. Extracellular matrix disturbance is caused by the degradation of collagen type IV, laminin, and fibronectin, as well as the loss of BBB integrity caused by active matrix metalloproteinases (MMPs), particularly MMP2 and MMP9, and other proteases released by neurons, activated glial cells, and endothelial cells [44, 45]. Several inflammatory cytokines have been involved in the pathogenesis of ischemic stroke, including IL-1b, IL-6, IL-18, and TNF. Proinflammatory cytokines produced and released by infiltrated leukocytes as well as activated cells in the brain parenchyma, such as neurons, oligodendrocytes, astrocytes, microglial cells, and microvascular endothelial cells, can cause neuronal cell death [46].

Recent research has shed light on further molecular mechanisms of neuronal injury in stroke, such as complement cascade activation, inflammasome activation, and hypoxisome activation [24]. Intrinsic risk markers of neuronal and glial cell membrane receptors may be activated by death-associated molecular patterns (DAMPs) generated by necrotic cells in the ischemic core, potentially leading to postischemic inflammation and neuronal cell death [47]. The synthesis of several complement components, including C1, C3a, and C5a anaphylatoxins, which are implicated in postischemic inflammation in neurons and glial cells, has been linked to the activation of the complement cascade and ischemic brain injury [48, 49]. Large multiprotein complexes called inflammatory cytosomes are present in the cytoplasm and are responsible for cleaving pro-caspase-1 into active cleaved caspase-1, which in turn cleaves pro-IL-1b and pro-IL-18 into mature forms that are released into the extracellular environment [24]. Additionally, cleaved caspase-1 can induce both pyroptosis and apoptosis [50, 51].

Stroke is an age-associated vascular disease. Obesity and hyperinsulinemia are risk factors for stroke. Chronic inflammation in obesity promotes vascular events, and studies have shown that thrombolytic therapy is less effective in obese patients. Caloric restriction diet may be an efficient strategy to accelerate improvement in neurological deficits in ischemic stroke. Ciobanu et al. [52] reported that calorie restriction decreased serum insulin growth factor 1 (IGF1), insulin, free fatty acid, and cerebral infarction volume using anti-NeuN antibody in aged animals with the middle cerebral artery occlusion (MCAO) model. Caloric restriction diet induced motoric improvement and spatial reference memory between 7 and 14 days compared to the control group and upregulated neuroprotective genes like *Prkaac/Prkga1* and *Igf2* and *Mapkapk2* genes that support angiogenesis. Studies on mesenchymal stromal cell–derived small extracellular vesicles (MSC-sEVs) demonstrate their potential to promote neurological recovery and brain remodeling in aged rats after stroke, highlighting the importance of this evaluation. Specifically, MSC-sEVs have been shown to reduce motor coordination deficits and macrophage infiltration in aged rats, suggesting a shift toward a more tolerogenic immune response and enhanced angiogenesis and neurogenesis [53].

Numerous strategies have been developed for the treatment of ischemic stroke aimed at enhancing neuroprotection in the brain, one of which involves the administration of natural medicines such as MLC 901. Various pathomechanisms associated with acute ischemic stroke represent critical targets for natural therapeutic approaches. Natural bioactive components, whether in combination or as extracts, have the potential to mitigate oxidative stress induced by stroke, reduce neuroinflammation, modulate microglial polarization, regulate energy metabolism, diminish neuronal excitotoxicity, and preserve the integrity of the BBB [15]. Astragaloside, salvianolic acid B, paeoniflorin, and ferulic acid are the most frequently studied proangiogenic effects. Astragaloside induces activation of the miRNA-210-mediated hypoxia-inducible factor (HIF)-1/vascular endothelial growth factor (VEGF)/Notch pathway in MCAO rats. *Salvia miltiorrhiza* extract promotes the proangiogenic process with upregulation of VEGF, brain-derived neurotrophic factor (BDNF), and endothelial nitric oxide synthase (eNOS) expression in the peri-infarct region. Paeoniflorin has angiogenic action through the upregulation of VEGF/VEGF receptor-2 expression [54].

### 3.2. The Effect of Nine Herbal Components of MLC 901 in Cerebral Ischemia

MLC 901 is a traditional medicine approved by the Chinese Food and Drug Administration in 2001 as a drug used by patients after stroke to improve neurological function [55, 56]. There are nine herbal components contained in MLC 901, including Radix astragali, Radix salvia miltiorrhizae, Radix paeoniae rubra, Rhizoma chuanxiong, Radix angelicae sinensis, *Carthamus tinctorius*, *Prunus persica*, Radix polygalae, and Rhizoma acori tatarinowii [16, 57, 58]. This herbal component contains various active compounds such as astragaloside IV (AST-IV), total paeony glycoside (TPG), tanshinone IIB (TSB), salvianolic acid B (SAB), tetramethylpyrazine (TMP), ligustilide and butylidenephthalide, ferulic acid, *β*-asarone, presenegenin, and hydroxyl safflower yellow A (HSYA) [57–59].

Various pharmacological studies show that MLC 901 has a neuroprotective effect on neurons suffering from ischemic damage, thereby leading to improved cognitive function and poststroke recovery [18, 55]. This effect is achieved through stimulation of cell proliferation, synaptogenesis, neuronal growth, and prevention of hippocampal damage from global ischemic injury [55]. Rosyidi et al. [60] reported that the administration of MLC 901 improves synaptophysin, a marker of neurogenesis. The increase of synaptophysin antibody in the cytoplasm of nerve cells indicates the neurogenesis process, gliogenesis, improves brain remodeling, neuronal plasticity, and correlates with functional recovery [60]. The herbal components and active compounds in MLC 901 work synergistically to enhance poststroke recovery. It is because each component can target different mechanisms. TSBs, ferulic acid, *β*-asarone, and AST-IV have been shown to have therapeutic effects by reducing BBB permeability, infarct volume, and nerve damage, as well as correcting neurological deficits in ischemic stroke models [61–66].

The primary effects of various active compounds, including AST-IV, SAB, TSB, TMP, ferulic acid, ligustilide, butylidenephthalide, *β*-asarone, HSYA, TPG, and presenegenin, involve the inhibition of apoptotic and inflammatory pathways. These mechanisms play a crucial role in neuroprotection during ischemic stroke, as they contribute to the preservation of neuronal integrity and function under conditions of reduced blood flow [16, 62, 67]. MLC 901 specifically targets the apoptosis pathway linked to caspase-3, which is associated with mitochondrial function. Notably, Z-ligustilide, an active component found in Radix angelicae sinensis, has been shown to significantly decrease the expression levels of both caspase-3 and Bax proteins within the ischemic cortex. This reduction is instrumental in inhibiting the apoptotic process, thereby contributing to neuroprotection in ischemic conditions [68]. AST-IV contained in Radix astragali can protect postischemic–reperfusion injury neurons by activating the epidermal growth factor receptor/nuclear factor erythroid-2-related factor 2 (EGFR/Nrf2) signal pathway. Activation of this signal pathway can also repair the damaged neurons and improve the regulation of nerve growth factor messenger ribonucleic acid (NGF mRNA) shortly after the stroke [69, 70]. *Carthamus tinctorius* extract has been shown to inhibit apoptosis to minimize ischemic–reperfusion injury by reducing the levels of Bax and caspase-3 proapoptosis, while increasing levels of B cell lymphoma 2 (Bcl-2) and mRNA antiapoptosis [71]. The HSYA contained in *Carthamus tinctorius* also shows anti-inflammatory, antioxidant, and antithrombotic effects on ischemic stroke models [72–74]. Radix polygalae extract shows significant neuroprotective effects against apoptosis and oxidative stress through increased expression of Nrf2 and heme oxygenase (HO)-1 in RAW 264.7 mouse macrophages cells induced by lipopolysaccharide (LPS), while improving neurogenesis, neuronal growth, neuron plasticity, and neurotransmitter reuptake [75, 76]. Radix polygalae can also inhibit glutamate release, inhibit increased intracellular levels of Ca^2+^, inhibit the death of nerve cells induced by NMDA (1 mM), and inhibit rats' brain granule neuron formation [77, 78].

The combination of Radix paeoniae rubra and Rhizoma chuanxiong has been shown to reduce the size of infarction in the brain of rats after MCAO by reducing the levels of IL-1*β*, IL-6, IL-12, and IFN-*γ* in the serum and brain tissue of MCAO rats [79, 80]. *β*-Asarone contained in Rhizoma acori tatarinowii can reduce cerebral autophagy due to ischemic reperfusion in rats through modulation of Bcl-2 and Beclin 1, phospho-JNK (p-JNK), and c-jun N-terminal kinases (JNK) [81]. The TPG contained in Radix paeoniae rubra has a role in restoring energy metabolism by increasing the activity of Ca^2+^-ATP and Na^+^-K^+^-ATP enzymes in the model of cerebral ischemic reperfusion injury [17].

In conditions of occurrence of neuroinflammation after ischemic stroke, TMP contained in Rhizoma chuanxiong shows neuroprotective effects by impeding the activation, migration, and aggregation of inflammatory cells such as astrocytes and microglia [62, 67]. The SABs contained in Radix salvia miltiorrhizae show neuroprotective effects by blocking Toll-like receptor-4 (TLR4) signaling pathways in MCAO-induced ischemic stroke models. In addition, these blockages can suppress nuclear factor kappa beta (NFkB) transcription and proinflammatory cytokine responses such as IL-1*β*, IL-6, and TNF-*α* [80]. The ferulic acid found in Radix angelicae sinensis has also been shown to suppress NFkB phosphorylation in the brains of infarction rats [82]. The polyphenols and carotenoids contained in *Prunus* peach have antioxidant effects. Both of these also have a decreasing regulatory effect on chemokine 4 (CCL-4) ligands that can inhibit IL-1*β*, TNF-*α*, NFkB, and RAGE expression in CCL-4-induced inflammatory rats [83].

Potent angiogenic factors are also one of the potential targets of MLC 901. Dl-3n-butylphthalide contained in Radix angelicae sinensis and Rhizoma chuanxiong has a role in protecting brain tissue from damage during the occurrence of ischemic stroke by controlling VEGF and HIF-1 alpha expression [84]. Wahyudi et al. reported that the administration of MLC 901 for 14 days can reduce VEGF mRNA expression, which indicates a neuroprotective effect where reducing VEGF levels can decrease cell damage and reduce brain edema and infarction size [85]. The various herbal components and active compounds contained in MLC 901 make it effective as a therapeutic intervention for stroke patients. The key is to ensure an effective combination of herbal extracts and the interaction between the components [16].

The AST-IV showed inhibition of TLR and NFkB, thereby reducing TNF-*α* and MMP-9 levels [86]. The active ingredient SAB inhibits ischemia–reperfusion through upregulation of heat shock protein (HSP) [87]. MLC 901 inhibits neutrophil infiltration into the BBB, inhibiting MMP-9 release and reducing brain edema [58]. In addition, MLC 901 reduces glutamate levels so that ROS levels decrease, thus inhibiting necrosis and apoptosis and improving neurological severity [57].

### 3.3. Molecular Mechanism of MLC 901 in Acute Ischemic Stroke

MLC 601 or 901 is a traditional Chinese medicine that has proven to possess neuroprotective and neuroregenerative properties in conditions such as stroke, global cerebral ischemia, and traumatic brain injury [88]. According to recent research, MLC 601 is an effective treatment for motor recovery in patients with ischemic stroke; the study showed that MLC 601 performed better than placebo and was safe to use in conjunction with standard medications for ischemic stroke, particularly in severe and moderate cases. These findings suggest that MLC 601 could be a valuable addition to the treatment options available for ischemic stroke patients [88]. Previous studies on mice administered MLC 901 for 5 weeks before ischemia and during the recovery process have shown its ability to protect mice from brain injury caused by focal cerebral ischemia. This resulted in a reduced brain damage area and hemisphere swelling while also improving the survival rate of mice after ischemic stroke. Additionally, the infarction volume of the rats' brains following MCAO was compared to those of rats treated with MLC 901 for 5 weeks before MCAO induction and mouse killing for analysis. Compared to the group treated with a vehicle, the group provided MLC 901 had a reported total infarct volume that was smaller. Furthermore, the treatment group had noticeably less cerebral edema than the vehicle group. This is in line with the concept that MLC 901 increases BDNF to enhance neurogenesis and synaptogenesis, consequently limiting more brain damage and promoting brain plasticity and eventual neurorepair following brain injury [89] (Table 1).

There are multiple suggested mechanisms that could explain the potential advantages of MLC 601 or MLC 901 in safeguarding against ischemic harm. Recent research indicates that these substances have the ability to boost the expression of BDNF, a protein that is essential for promoting growth, differentiation, and potentially supporting neurogenesis [101, 102]. MLC 901 was demonstrated to stimulate neurogenesis in the dentate gyrus's subgranular zone, as evidenced by the increased quantity of neural precursors seen in an ischemic rat model [10]. Furthermore, MLC 901 was shown to elevate (upregulate) the growth-associated protein (GAP43) in cortical neurons treated with the compound, suggesting that it may promote neurite development with a denser neurite network [57]. Collectively, these actions may contribute to the neuroprotective effects of MLC 601 or MLC 901 against ischemic injury. Neurons are highly susceptible to damage caused by oxygen deprivation (hypoxia) and overstimulation (excitotoxicity). A model of oxygen–glucose deprivation (OGD) has demonstrated the effectiveness of MLC 901 in mimicking the cell death processes observed in the salvageable (penumbral) regions of the brain during ischemia. When cortical neurons were deprived of oxygen and glucose for 2 h, they immediately swelled and calcium influx was observed during the early phase of reoxygenation. Subsequently, neuronal degeneration occurred over the next 24 h, along with the release of lactate dehydrogenase (LDH) into the surrounding media. MLC 901 was found to reduce OGD-induced calcium influx and excitotoxicity in neurons [103].

MLC 901 has been shown to increase animal survival, function recovery, and decrease neurodegeneration in rats' brain ischemia models [57]. Research has indicated that MLC 901 may have a neuroprotective effect that is associated with a significant hyperpolarization process. This process can be impeded by glibenclamide, a specific inhibitor of ATP-sensitive K^+^ channels (KATP). Experiments performed on mouse cortical neurons have demonstrated that MLC 901 effectively activates KATP channels, similar to the typical KATP channel opener, pinacidil [102]. The activation of KATP channels via MLC 901 results in hyperpolarization, which can help prevent the substantial release of excitotoxic glutamate and glutamate-triggered Ca^2+^ influx, particularly in neurons that have undergone energy deprivation, for a brief period [101]. Anjum et al. [90] found that therapy improved cell survival and encouraged neural regeneration, supported by the greater expression of p53 seen in the treated cells, which was found to be lower in the untreated cells. Additionally, compared to the untreated cells, this study demonstrated a significant rise in GAP43 expression, suggesting that NSC-34 cells were at a higher stage of regenerative activity. MLC 901 treatment resulted in an increase in eIF2*β* expression compared to untreated cells, suggesting the regeneration of neurons. Since ATF-3 is a sign of active regeneration, higher production of it in MLC 901-treated cells guaranteed the cells' capacity for neuroregeneration. In treatment groups, there was a downregulation of p-GSK3*β* and an increase in the expression of all survival markers, including p-AKT, GAP43, ATF-3, p53, and eIF2*β* [90].

MLC 901 has been shown to be beneficial in reducing the expression of proinflammatory mediators caused by a stroke. Notably, at 24 h after ischemic insult, a significant increase in neutrophil infiltration was seen in the damaged cortex of mice treated with vehicle [58]. However, MLC 901 therapy reduced neutrophil infiltration significantly, implying that this drug can reduce neuronal damage induced by stroke by blocking detrimental neutrophil recruitment [58]. During the initial phase of stroke, infiltrating neutrophils cause extreme production of ROS, which leads to oxidative stress in the injured brain tissue. This oxidative stress can activate NF-*κ*B, which regulates the transcriptional induction of various proinflammatory genes [104, 105]. Oxidative stress plays a part in the development of infarction and contributes to increased damage in the affected area [104]. However, Widmann et al. found that MLC 901 significantly reduced the level of phosphorylated NF-*κ*B, which is a marker of NF-*κ*B activation. This inhibition of neutrophil recruitment by MLC 901 could lead to a decrease in NF-*κ*B activation and inflammation via the NF-*κ*B pathway; this is because high levels of ROS are well known to be produced by recruited neutrophils. MLC 901 has a protective effect against brain damage by decreasing mRNA expression of IL-6, IL-11, IL-1, and TNF-*α* [58]. In addition, the neuroprotective effect of MLC 901 by modulating various signal pathways such as the phosphatidylinositol-3-kinase (PI3K) pathway, MAPK pathway, cAMP response element-binding protein (CREB) pathway, neurotrophic factor (NTF), and nuclear factor erythroid factor-2-related factor-2 (Nrf2) is notable. Modulation of all signal pathways results in decreased oxidative stress, apoptosis, infarct volume, neuroinflammation, neuronal cell injury, and improved motoric function [106].

## 4. Conclusions

Ischemic stroke can cause severe and permanent damage to cells in the brain. The damage is caused by various pathways working together. MLC 901 is a form of traditional Chinese medicine that has been proven to have neuroprotective and neurorestorative properties, which makes it useful in treating conditions such as stroke, global cerebral ischemia, and traumatic brain injury. MLC 901 works by enhancing the expression of BDNF, a protein that plays a crucial role in promoting growth and differentiation, and may also facilitate neurogenesis. Additionally, MLC 901 can activate KATP channels, which further helps in the treatment of ischemic stroke.

## Figures and Tables

**Table 1:** Experimental studies of MLC 901 in stroke.

**Studies**	**Methods**		**Outcomes**
	**Sample**	**Intervention**	
Gandin et al. [89]	A total of 378 animals (4-week-old C57BL/6 male mice)	A stroke model was induced by 60 min of middle cerebral artery occlusion followed by reperfusion. MLC 901 was administered to the animals via their drinking water (6 g/L) for 5 weeks before the onset of ischemia and then continued during reperfusion.	Improved survival rate after ischemia and reperfusion, while also reducing the area of cerebral infarction. MLC 901 treatment was effective in preventing blood–brain barrier leakage and reducing neurological dysfunction.
Heurteaux et al. [57]	Adult male C57/Bl6 mice	In vitro experiments: A concentration of 1 mg/mL was used in each 24-mm well. MLC 601 or MLC 901, contained in a capsule of 400 mg, was diluted in 40-mL neurobasal medium, resulting in a concentration of 10 mg/mL (stock solution) at 37°C for 60 min. Cell treatment with MLC 601 or MLC 901 began on Day 3 of culture and lasted for 14 days (equivalent to 17 days of culture). In vivo experiments: MLC 901 pretreatment was administered in drinking water at a concentration of 6 mg/mL. One capsule of MLC 901 was dissolved in 66-mL water with stirring for 1 h at 37°C, followed by filtration with a 0.22-mm filter. For in vivo posttreatment, mice received a single intraperitoneal injection of either MLC 901 or MLC 601 solution at a concentration of 2 mg/mL, diluted in saline (as vehicle). This injection was administered to mice weighing 25 g, with a total volume of 500 mL, at two time points: At the beginning of ischemia and 6 h after reperfusion (referred to as posttreatment onset) or at 3 and 24 h following the end of ischemia (referred to as posttreatment 3H). The pretreatment dosage for in vivo experiments was determined based on the concentration used in humans (four capsules thrice a day for oral administration).	Enhances survival and protects the brain from ischemic injury and significantly decreases functional deficits. MLC 601 and MLC 901 have also demonstrated the ability to prevent neuronal death in an in vitro excitotoxicity model employing primary cultures of cortical neurons exposed to glutamate. Furthermore, treatments involving MLC 601/MLC 901 have been observed to trigger neurogenesis in both rodent and human cells, foster cell proliferation, facilitate neurite outgrowth, and encourage the formation of a dense axonal and dendritic network.
Widmann et al. [58]	Seven-week-old C57BL/6 male mice (Janvier)	A single dose of 40 *μ*g/kg of MLC 901 was administered intraperitoneally. MLC 901 is diluted in saline (as a carrier) 90 min after the onset of ischemia and once a day during reperfusion. C57BL/6 male mice (Janvier) were used to model an ischemic stroke by subjecting them to middle cerebral artery occlusion (MCAO) for 60 min and then reperfusion.	The administration of MLC 901 resulted in decreased blood–brain barrier leakage and brain swelling, decreased infarction volume, enhanced neurological scores, and decreased mortality at 24 h after middle cerebral artery occlusion (MCAO).
Wahyudi et al. [85]	10 male Sprague-Dawley rats	Before receiving treatment, the 250–300 g Sprague-Dawley rats were allowed to acclimate for 1 week. The treatment group consisted of five male Sprague-Dawley rats (T). The control group consisted of five male Sprague-Dawley rats (C). At a concentration of 75 mg/mL, MLC 901 was diluted in saline (as a vehicle) and incubated for 1 h at 37°C with agitation. Rats were administered a single intraperitoneal injection of MLC 901 at a dosage of 0.075 mg/mL (in a 500-mL bolus) 2 h after spinal cord injury (SCI). Ten milligrams of the drug was then orally administered in drinking water until the animals were sacrificed.	The administration of MLC 901 for 14 days can reduce vascular permeability by reducing VEGF mRNA expression, which indicates a neuroprotective effect where reducing VEGF levels can decrease cell damage and reduce brain edema and infarction size.
Anjum et al. [90]	Motor neurons isolated from NSC-34 cells	This study used an in vitro injury model with mechanical scratches. After being mechanically scratch-wounded, motor neurons isolated from NSC-34 cells were analyzed to determine MLC 901's capacity for neuroregeneration in both treated and untreated cells. NSC-34 differentiation into motor neurons (MN) was facilitated by retinoic acid (RA) (1 and 10 *μ*M) and 30 *μ*M prostaglandin E2 (PGE2) induction. Different concentrations of NeuroAiD II (MLC 901) were applied to the MN after it had been scratched. The AKT/P13K pathway analysis, immunocytochemistry (ICC), and time-lapse experiment were carried out. Initial concentration of 50 mg/mL of MLC 901 was added to double-distilled water, vortexed for 1 min, and incubated for 1 h at 37°C. Before cell treatment, the mixture was centrifuged for 3 min at 1000*g* and passed through a 0.22-*μ*m filter. MLC 901 at several doses (25–2000 *μ*g/mL) was applied to the differentiated NSC-34 cells for 2 days, for a total of 16 h.	MLC 901 has been proven to be a successful neuroregenerative and neuroprotective treatment for SCI. The tremendous potential of the scratch injury model to serve as a prototype screening method for promising nanotherapeutic interventions. MLC 901 at 1000 and 1200 *μ*g/mL significantly increased neurite outgrowth in comparison to untreated cells (UT). At doses of 1200 *μ*g/mL, the AKT/PI3K pathway analysis revealed greater expression of regeneration markers (p-AKT, p-GSK3*β*, ATF-3, GAP43, p53, and elF2*β*) compared to UT.
Pakdaman et al. [91]	35 patients with hypoxic–ischemic brain injury (HIBI)	In a randomized, placebo-controlled trial, 35 patients with HIBI were assigned to receive either MLC 901 or placebo capsules three times daily for 6 months. The randomization has been classified based on gender, age, and Glasgow Coma Scale. MLC 901 or matching placebo was taken orally with 2 capsules (vegetable capsules filled with < 2 g of stevia-sweetened powder) 3 times daily for 6 months. They examined participants at baseline and at 1, 3, and 6-month follow-up visits with a modified Rankin Scale (mRS) and Glasgow Outcome Scale (GOS).	The analyzed population for efficacy includes all 31 patients who completed the trial. A significantly higher improvement in the MLC 901 group compared to the placebo group is observed on both scales at Months 3 and 6 on mRS and from Months 1 to 6 with GOS. Overall, the functional improvement over time was significantly different between the 2 groups (repeated measures *t-*test: mRS *p* value: 0.021 and GOS *p* value: 0.018). There were no major adverse events related to the study treatment. Liver, renal function, and coagulation profile remained within the normal range in all patients in both groups.
Li et al. [92]	A total of 9 male Sprague-Dawley rats weighing between 230 and 260 g	Intranasal administration of Z-ligustilide (Z-LIG) and various surgical interventions were conducted in this study. Specifically, rats received intranasal pretreatment with Z-LIG at a dosage of 15 mg/kg for 3 days prior to undergoing MCAO surgery, a model that simulates the most prevalent form of stroke in humans. The MCAO procedure involved anesthetizing the rats and occluding the right middle cerebral artery using a silicone-coated nylon suture, followed by reperfusion after 1 h. Furthermore, lentiviral vectors encoding HSP70 were administered into the lateral ventricle of the ischemic hemisphere 5 days prior to the MCAO surgery to assess their impact on ischemic injury.	Intranasal pretreatment with Z-ligustilide (Z-LIG) significantly reduces infarct volume and neurological deficits following cerebral ischemic injury. The results indicate a marked decrease in TUNEL-positive cells, reflecting a protective effect against apoptosis due to MCAO. Additionally, Z-LIG mitigates BBB disruption and reduces brain edema, highlighting its neuroprotective properties. These findings suggest that Z-LIG enhances resilience to ischemic injury through the activation of cellular stress response pathways, particularly Nrf2 and HSP70.
Mahmood et al. [93]	A total of 24 mice divided into four groups: A sham group, a vehicle group, and two treatment groups receiving salvianolic acid (SAA) at doses of 1 and 5 mg/kg body weight	SAA was administered to male C57BL/6 mice, divided into four groups: A sham group, a vehicle group, and two treatment groups receiving SAA at doses of 1 and 5 mg per kg body weight. SAA or saline was given intragastrically for 7 days prior to a 60-min MCAO. Following MCAO, assessments included neurological evaluations and Nissl staining to measure brain infarct volume, as well as immunoblotting to examine the effects of SAA on calpain activation and eNOS uncoupling in the penumbral region.	Administration of SAA significantly improved glucose metabolism and reduced neuronal damage, as evidenced by decreased neurological deficits and infarct volume. SAA pretreatment effectively inhibited eNOS uncoupling and calpain activity, key factors in ischemic injury. The study also found that SAA decreased peroxynitrite production and increased phosphorylation of AKT, FKHR, and ERK, indicating a multitarget mechanism of action. These findings support SAA as a promising therapeutic strategy for neurovascular protection during cerebral ischemia, primarily by enhancing glucose metabolism and reducing ischemic damage.
Cheng et al. [94]	A total of 30 adult male Sprague-Dawley rats, 8 weeks old and weighing 300–350 g, were used in this study	This study employed various experimental protocols involving different groups of rats. In Experiment C, rats were divided into four groups: DS + sham, DS + control, DS + DG-1 g, and SB20 + DG-1 g. The SB20 + DG-1 g group received SB203580, a specific p38 MAPK inhibitor, prior to bilateral common carotid artery occlusion (BCCAO). The DS + DG-1 g group was treated with 1% dimethyl sulfoxide (DMSO) before BCCAO, while the DS + control group received DMSO without occlusion. In Experiment D, rats were randomly assigned to six groups: Sham, control, DG-0.25 g, DG-0.5 g, DG-1 g, and SB20 + DG-1 g. These groups underwent similar procedures as in Experiments A and C, with varying doses of DG administered. DG was administered intragastrically at doses of 0.25, 0.5, and 1 g/kg, starting 1, 3, and 5 days after transient global cerebral ischemia (GCI). The control group received saline instead of DG, while the sham group did not undergo occlusion. These interventions are aimed at assessing the neuroprotective effects of *Angelica sinensis* extract against ischemic injury in the hippocampus.	Treatment with *Angelica sinensis* extract at doses of DG-0.5 g and DG-1 g effectively protected hippocampal CA1 neurons from global cerebral ischemia/reperfusion (I/R) injury by activating the p38 MAPK/p90RSK signaling pathway. This activation helps preserve mitochondrial integrity and prevents oxidative stress–induced apoptosis via cytochrome c and caspase-3. Additionally, the neuroprotective effects are mediated through the CREB/BDNF signaling pathway, suggesting their potential as a therapeutic strategy for the subacute phase of global cerebral ischemic injury.
Seo et al. [95]	This study utilized the aerial parts (leaves, fruits, and twigs) of *Prunus persica* (peach tree) sourced from Bằng Lũng, Chợ Đồn, and Bắc Kạn in Vietnam. Six Sprague-Dawley rats (three males and three females) were used to prepare primary astrocytes, isolated from the cortices of Postnatal Day 2 rats.	The intervention methods in this study involved applying the methanol extract of *Prunus persica* (PPB) to BV2 cells and primary astrocytes. Primary astrocytes were treated with PPB at concentrations of 20, 100, and 200 *μ*g/mL for 1 h before exposure to lipopolysaccharide (LPS) at 10 ng/mL, while the control group received a vehicle (DMSO, 0.1%). BV2 cells were treated with LPS for 6 and 24 h for reverse transcriptase polymerase chain reaction (RT-PCR) and western blot analyses, respectively. The MTT assay was also used to assess cell viability after 24-h treatment with PPB.	The study concludes that the methanol extract of *Prunus persica* (PPB) exhibits significant anti-inflammatory effects in LPS-stimulated glial cells. PPB was shown to reduce the expression of proinflammatory mediators and cytokines at the transcriptional level, associated with the suppression of NF-*κ*B and MAPK activation in microglial cells. Additionally, PPB effectively inhibits nitric oxide production and p65 translocation in primary astrocytes, highlighting its potential as a therapeutic agent for neuroinflammatory and neurodegenerative diseases. The anti-inflammatory effects of PPB are likely attributable to its bioactive compounds, such as chlorogenic acid and catechin, known for their antioxidant properties.
Li et al. [96]	The study included 65 patients, with 56 completing the 12-week treatment. Participants were divided into two groups: The Astragali Radix (AR) group, consisting of 35 patients, and a control group. Each group had six biological replicates for proteomics analysis, equally comprising three men and three women.	A randomized, double-blind, placebo-controlled clinical trial was conducted to evaluate the efficacy of AR in treating cerebral infarction (CI). Patients were divided into an AR treatment group and a control group for 12 weeks. Therapeutic effects were assessed using traditional Chinese medicine (TCM) syndrome scores and clinical indicators, including the NIH Stroke Scale and Barthel Index. Serum samples from both groups were also analyzed using proteomics to explore the neuroprotective effects of AR.	This study demonstrates that Astragali Radix (AR) significantly alleviates the clinical symptoms of cerebral infarction (CI). Treatment with AR resulted in the upregulation of 43 proteins and downregulation of 20 proteins, indicating its potential neuroprotective effects. Furthermore, AR may influence inflammatory markers such as IL-6, TNF-*α*, intercellular adhesion molecule 1 (ICAM-1), vascular cell adhesion molecule 1 (VCAM-1), and monocyte chemoattractant protein 1 (MCP-1), which could contribute to its antiatherosclerotic and neuroprotective properties. Overall, this research provides a molecular basis for the clinical management of CI and elucidates the biological mechanisms through which AR exerts its effects.
Zhao et al. [97]	The sample of this study consisted of healthy male Sprague-Dawley (SD) rats, aged 10–12 weeks and weighing between 220 and 250 g	The intervention method in this study involved administering Paeoniae Radix Rubra extract (PRRE) and Buyang Huanwu Decoction (BYHWD) in a rat model of middle cerebral artery occlusion (MCAO). Healthy male Sprague-Dawley rats were randomly divided into six groups: sham-operated, MCAO, MCAO + PRRE (low, medium, and high doses), and MCAO + BYHWD. PRRE was given at doses of 0.27, 0.54, and 0.81 g/kg/day, while BYHWD was administered at 12.8 g/kg/day. Treatments were delivered via oral gavage daily for 3 days before and after surgery.	The study concluded that PRRE effectively reduces neurological deficits and infarct volume in a rat model of MCAO, thereby mitigating cerebral ischemic injury. Treatment with PRRE improved neuronal morphology and organization, indicating protective effects against damage in the cortex and hippocampus. Additionally, PRRE alleviated oxidative stress by modulating ferroptosis-related cytokines and activating autophagy via the PI3K/Akt signaling pathway, highlighting its potential as a therapeutic agent for cerebral ischemia.
Jiang et al. [77]	Various animal models, including rats and mice, were utilized to assess the effects of Radix Polygalae (RP) on neurological conditions	This study examined the effects of oral administration of Radix Polygalae (RP) extracts on neurological conditions. Results showed that RP at a dose of 200 mg/kg significantly reduced immobility in the tail suspension test (TST) and forced swim test (FST). Various RP fractions, including DISS and tenuifoliside A (TEA), were also evaluated for their antidepressant-like effects at dosages of 0.1 mg/kg and from 0.5 to 1 g/kg. Additionally, the extracts were assessed in behavioral paradigms to determine their impact on behavioral despair and anxiety-like behaviors in animal models.	This study demonstrates that Radix Polygalae has significant neuroprotective effects and potential therapeutic benefits for neurological disorders, such as Alzheimer's and Parkinson's diseases. The review confirms RP's well-established pharmacological properties, which impact various aspects of neurodegenerative and neuropsychiatric conditions. Key mechanisms include antioxidant effects, anti-inflammatory actions, and the promotion of neurogenesis and synaptic plasticity. The study also emphasizes the need for further investigation into the molecular and cellular mechanisms underlying RP's therapeutic potential.
Lim et al. [98]	At least five mice per group were used as the sample of this study	This study investigated the neuroprotective effects of an orally administered methanolic extract of Chuanxiong Rhizoma (CRex) in experimental mice. The effects of CRex on cerebral infarction, cognitive deficits, and behavioral functions were evaluated through various assessments. Mice received different dosages of CRex prior to the induction of MCAO to assess its impact on neurological deficits, forepaw grip strength, and cognitive function. Behavioral evaluations, including the novel object recognition test (NORT), were conducted to measure the mice's ability to recognize familiar versus novel objects. Comprehensive evaluations included monitoring body weight, assessing physiological parameters, collecting blood samples, and analyzing plasma electrolyte levels to assess the effects of CRex in MCAO-induced cerebral infarction.	The study found that pretreatment with Chuanxiong Rhizoma extract (CRex) at doses of 1000 and 3000 mg/kg significantly reduced cerebral infarction after MCAO. However, CRex did not significantly inhibit cerebral edema, indicating limitations in its protective effects against certain stroke damage. While it did not alleviate neurological deficits, CRex mitigated the reduction in forepaw grip strength, suggesting partial protection of motor function. Additionally, CRex treatment significantly improved cognitive deficits induced by MCAO, indicating a positive impact on cognitive recovery poststroke.
Wen et al. [99]	Three samples were used in the study: AD mice, HT-22 cells, and double transgenic mice expressing the APP/PS1 to model AD	This study investigated the efficacy of Smart Soup (SS), a traditional Chinese medicine formulation containing ATR, PRP, and PR, in Alzheimer's disease (AD). SS was administered orally to AD transgenic mice to evaluate its effects on cognitive impairment, A*β* levels, A*β* amyloidosis, brain gliosis, and neuronal loss. Additionally, SS treatment significantly reduced amyloid-related motor dysfunction and premature mortality in AD transgenic flies, underscoring its potential neuroprotective effects and therapeutic role in AD.	The study concludes that Acori Tatarinowii Rhizoma (ATR) has a rich history of medicinal use and exhibits a wide range of pharmacological effects relevant to central nervous system disorders, such as depression, Alzheimer's disease, Parkinson's disease, epilepsy, and cerebral ischemia–reperfusion injury. Its multicomponent, multitarget, and multipathway synergistic properties make ATR a promising candidate for future drug development due to its diverse mechanisms of action.
Ko et al. [100]	The sample of this study consisted of mice as the experimental subjects. The study utilized a total of 3–4 mice per group for the analysis	This study utilized a 28-day repeated administration of SM (a specific substance) following transient middle cerebral artery occlusion (tMCAO) in murine models. The intervention's efficacy was assessed by examining its effects on neurological deficits, survival rates, and cognitive impairments. Furthermore, the study investigated the therapeutic mechanisms underlying the long-term neuroprotective effects of the intervention in the tMCAO stroke model.	The study concludes that the administration of SM mitigated cerebral ischemia/reperfusion (I/R) injury and restored metabolite levels in the brains of mice subjected to tMCAO. SM treatment improved neurological deficits, reduced oxidative stress, and exhibited an antiferroptotic effect in the tMCAO model. Collectively, these findings indicate that SM holds potential for protecting against acute brain injury and enhancing outcomes in mice following MCAO and reperfusion.

## Data Availability

No underlying data was collected or produced in this study.
